# Current-voltage characteristics in macroporous silicon/SiO_x_/SnO_2_:F heterojunctions

**DOI:** 10.1186/1556-276X-7-419

**Published:** 2012-07-25

**Authors:** Felipe A Garcés, Raul Urteaga, Leandro N Acquaroli, Roberto R Koropecki, Roberto D Arce

**Affiliations:** 1, Instituto de Desarrollo Tecnológico para la Industria Química, UNL/CONICET, Güemes 3450, Santa Fe, S3000GLN, Argentina; 2, Facultad de Ingeniería Química,UNL, Santiago del Estero 2829, Santa Fe, S3000AOM, Argentina

**Keywords:** Macroporous silicon, Transparent conductor oxide, Spray pyrolysis, Electrical anodization

## Abstract

We study the electrical characteristics of macroporous silicon/transparent conductor oxide junctions obtained by the deposition of fluorine doped-SnO_2_ onto macroporous silicon thin films using the spray pyrolysis technique. Macroporous silicon was prepared by the electrochemical anodization of a silicon wafer to produce pore sizes ranging between 0.9 to 1.2 *μ*m in diameter. Scanning electronic microscopy was performed to confirm the pore filling and surface coverage. The transport of charge carriers through the interface was studied by measuring the current-voltage curves in the dark and under illumination. In the best configuration, we obtain a modest open-circuit voltage of about 70 mV and a short-circuit current of 3.5 mA/cm^2^ at an illumination of 110 mW/cm^2^. In order to analyze the effects of the illumination on the electrical properties of the junction, we proposed a model of two opposing diodes, each one associated with an independent current source. We obtain a good accordance between the experimental data and the model. The current-voltage curves in illuminated conditions are well fitted with the same parameters obtained in the dark where only the photocurrent intensities in the diodes are free parameters.

## Background

Fluorine-doped tin oxide SnO_2_:F(FTO) and porous silicon (PS) are two types of materials that have been extensively investigated for sensor applications [1,2]. Tin oxide is a transparent conductive oxide with electrical transport properties extremely sensitive to the environment [3-6]. Porous silicon is a material that can exhibit efficient visible photoluminescence [7,8], and several sensing applications using PS layers have been reported in, for example, humidity sensors [9], gas sensors [10-13], and biological sensors [14]. Since both materials, PS and FTO, exhibit an elevated specific surface, they are potentially attractive for these types of applications. It is expected that combining both materials in a single device will lead to an enhancement of their sensing properties. In this way, the current-voltage or capacitance-voltage characteristics in such materials are modified when these devices are subjected to altered environments [15-17]. In this work, we study the properties of the junctions made by FTO deposited on macroporous silicon. The development of this work should help in understanding the response of these heterojunctions to gaseous analytes. The progress in PS optoelectronics depends on the understanding of the operating principles of PS devices. However, little work has been done on the electrical transport of macro-PS device structures in comparison to the research done on the optical and electrical properties of nano-PS and meso-PS [13,18,19]. Up to now, several models have been proposed for the transport of carriers in PS-based metal/PS/c-Si device structures [20-23]. These reports explain the behavior assuming that reverse current is determined by the surface mechanism associated with hopping [21] or with carrier generation from the surface states on the boundary between PS and the c-Si substate [20]. The transport properties of oxidized (metal/PS/p-Si) structures have been hardly investigated, although relatively effective and stable electroluminescent device and photodetector structures based on oxidized PS were fabricated [20]. Typically, the PS layer is sandwiched between the c-Si substrate and a metallic contact. This contact is usually gold or aluminium. Not much is known about the interfaces since band alignment depends on PS electronic properties. Nevertheless, in most literatures, the PS layer was considered to behave like a wide band gap semiconductor and assumed a Schottky barrier formed between the metal and the PS. In some cases, these metal contacts are replaced by transparent conductive oxide (TCO), such as tin oxide [24] or zinc oxide [25], modified with dopants such as fluoride or aluminium, respectively. In these cases, knowledge about the contact properties is very scarce. In this work, we present the results obtained for metal/c-Si/PS/FTO and metal/c-Si/PS/SiO_x_/FTO heterojunctions. We measured the *J-V* characteristic in the dark and under illumination for the prepared junctions. The transport parameters were obtained by fitting the characteristic *J-V* curves. The effect of illumination on the heterojunctions and transport properties is discussed as well. Morphology characterization was completed with scanning electron microscopy (SEM).

## Methods

Porous silicon layers were obtained by electrochemical anodization of p-type boron-doped crystalline silicon wafers, with an orientation of (100) and resistivity of 10 to 20 *Ω* cm, in a hydrofluoric acid 50% and N,N dimethylformamide electrolyte solution in proportions of 1:9 in volume. The galvanostatic process was carried out for 1,800 s using a 10-mA/cm^2^ current density in darkness. A Teflon^®^anodization cell with platinum contact as the cathode and the silicon wafer as the anode was used. Aluminium 99.99% was evaporated as a backside contact of the Si wafer to improve the distribution of the current density in the anodization stage and to achieve an ohmic contact on the backside of the silicon substrate. Prior to the FTO deposition, some samples were oxidized in a rapid thermal annealing furnace. This oxidation was carried out at atmospheric pressure using a two-step process: (1) 450°C for 10 min followed by (2) 550°C for 30 min. The FTO, the n-type region in our devices, was fabricated starting from a synthesized precursor in order to get tin oxide by the sol-gel method [26]. Subsequently, with this precursor, we proceeded with the deposition of a layer of SnO_2_:F using a spray pyrolysis method [27]. The deposition temperature was set at 380°C, and it was controlled within ±2°C. The deposited thickness was about 900 nm with 20 min deposition. In this way, two types of heterojunctions were fabricated: Al/c-Si/PS/SnO_2_:F (abbreviated in the following as PS/FTO) and Al/c-Si/PS/SiO_x_/SnO_2_:F (abbreviated as PS/Ox/FTO). The *J-V* measurements were performed in a sandwich configuration using a Keithley 6487 digital picoammeter/voltage source (Keithley Instruments, Inc., Cleveland, OH, USA). The voltage was applied between the top FTO contact and the ohmic back contact. For forward bias, the ohmic back contact was grounded, and the FTO contact was biased negatively. The photoresponse of the device was measured by illuminating the sample with a halogen MR16 lamp with a light intensity of 110 mW/cm^2^. A set of optical density (OD) filters was used to obtain different illumination intensities from 110 mW/cm^2^ (OD = 0) to 1.10 × 10^−1^mW/cm^2^ (OD = 3). These measurements were made at 300 K. All layers were characterized by SEM in a JEOL J5M-35C microscope (JEOL Ltd., Akishima, Tokyo, Japan).

## Results and discussion

**Figure 1:**
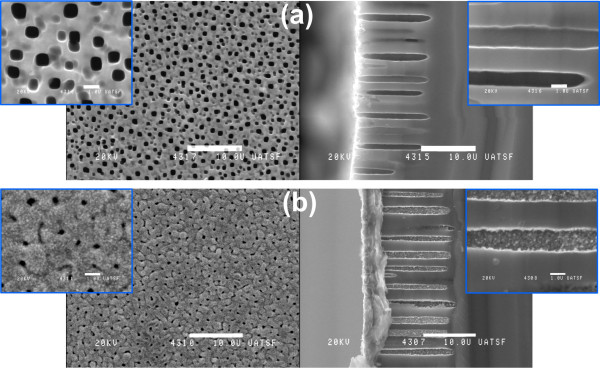
**SEM images. (a)** Without deposited FTO and **(b)** with deposited FTO. The white bars correspond to 10 *μ*m for the top view (left) and the profile (right) images.

Figure 1a, b exhibits representative SEM images of heterojunction diodes without and with the tin oxide layer deposited by spray pyrolysis, respectively. The PS layers with uniform tickness (15 *μ*m) have been fabricated by electrochemical anodization of Si wafers. The sprayed SnO_2_:F films were deposited on the surface of the PS layer and within the pores themselves. The process leading to the formation of SnO_2_:F grains on the external surface and inside the pores generates grain sizes of about 80 to 120 nm. These particles are present at the surface and within the porous structure.

**Figure 2:**
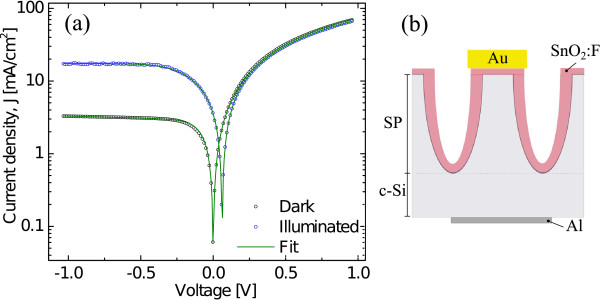
**PS/FTO *J-V *characteristics.** Current-voltage characteristics of PS/FTO heterojunctions. Dark and illuminated condition curves are shown. The dark condition characteristic of this heterojunctions presents a rectifying behavior in reverse bias, similar to that of a conventional diode. When illuminated at 110 mW/cm^2^, PS/FTO generates a photovoltaic effect with an the open-circuit voltage value of 70 mV and short-circuit current of 5 mA/cm^−2^. A fit with a simple diode model is also included.

Figure 2 shows the *J-V* characteristics of the heterojunction PS/FTO in the range −1 to +1 V in a semilog plot. The voltage was applied between a golden pin front contact and an aluminium back contact, as shown in the inset in Figure 2b. Devices with this configuration are usually rectifying due to the formation of interface barriers. The associated carrier transport mechanisms are similar to those observed in normal *p-n* heterojunction [28] showing a rectification ratio in darkness of approximately 20 at ±1 V. When the sample is illuminated, this heterojunction generates a photovoltaic effect, and the reverse current strongly increases for growing reverse voltage. The value of the open-circuit voltage is about 70 mV, and the short-circuit current is 3.5 mA/cm^2^ at an illumination of 110 mW/cm^2^. The experimental data presented in Figure 2 were fitted with a simple diode model [29]. As a result, we obtained the following set of parameters: series resistance (*Rs*), reverse saturation current (*I*_0_), the ideality factor (*n*) and the photogenerated current (*I*_*L*_). Although these *J-V* curves perfectly fit with the model of a simple diode both in the dark and under illumination, subtle changes in the fitting parameters occur when moving from one situation to another. Values in dark conditions are *n*=1*.*8, *I*_0_=2*.*9 mA/cm^2^, *Rs*=35*.*4 *Ω* and *Rp*=1*.*18×10^4^*Ω*. On the other hand, the parameters for the illuminated case are *n*=3*.*93, *I*_0_=9*.*33 mA/cm^2^, *Rs*=37 *Ω*, *Rp*=3*.*5×10^4^*Ω*. 

**Figure 3:**
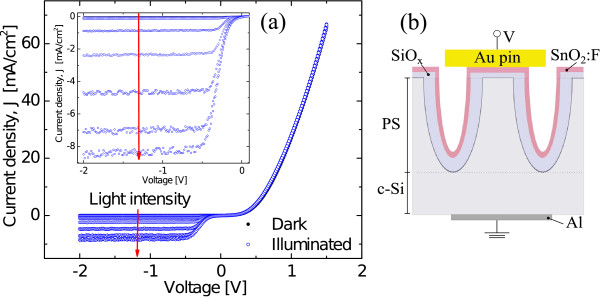
**PS/Ox/FTO junction *J-V *characteristic.** The current-voltage characteristics of PS/Ox/FTO heterojunctions with different light intensities (light intensity increases in the arrow direction) ranging from dark to 110 mW/cm^2^. Inset in **(a)** shows a detail of reverse bias region, and **(b)** shows the schematic configuration (sandwich type) of layers in the heterojunction.

Figure 3 shows the *J-V* characteristics of the heterojunction with the intermediate oxide layer PS/Ox/FTO under different light intensities. The voltage was applied between a golden pin front contact and an aluminium back contact, as shown in the inset in Figure 3b. The rectification ratio for the *J-V* characteristic curve obtained in darkness was 500 at ±1 V. In the figure are also shown the *J-V* characteristics for different light intensities.

It is possible to observe in the reverse bias region an increase in the photogenerated current when the light intensity is augmented. The current is significantly modified by the illumination only for reverse bias. The inset in Figure 3a shows a detail of the current behavior under illumination for the range between −2 and 0 V.

The current increases more than two orders of magnitude with respect to the current in dark condition. The junction has, in this case, an open-circuit voltage of 50 mV, similar to that shown in Figure 2, but the short-circuit current is 85 *μ*A/cm^2^ (almost two orders of magnitude lower than that in Figure 2) at illumination of 110 mW/cm^2^.

**Figure 4:**
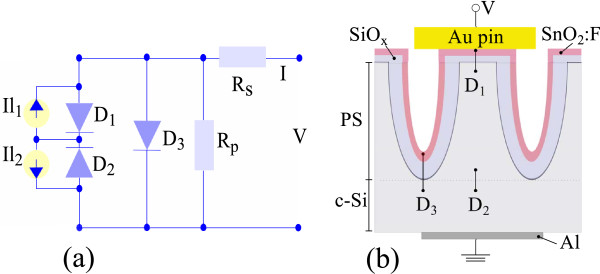
**Model of PS/Ox/FTO heterojunction.** Proposed equivalent circuit **(a)**. Two opposite diodes with photocurrent sources are connected in parallel to another diode. Parallel and series resistance are also included. Schematics of PS/Ox/FTO heterojunction and the interfaces associated to each diode in the equivalent circuit **(b)**.

The observed behavior in the reverse bias is similar to that observed in devices based on ZnO/Si [30,31]. In that case, it was attributed to a native SiO_x_ layer at the ZnO/Si interface acting as a double Schottky barrier for both n-type layers. In the forward bias region, the current does not change significantly with the illumination intensity and is similar to that measured in the PS/FTO heterojunction in Figure 2. On the bases of these observations, we propose a model consisting of two back-to-back diodes associated with two independent photocurrent sources in parallel with a simple diode. The model is presented in Figure 4, where a scheme of the heterojunction PS/Ox/FTO is shown. In this scheme, the *J-V* characteristic will be determined by the contribution of the two components connected in parallel. 

In the combination of back-to-back diodes, the current is limited for both polarities of the device. In this configuration, *D*_1_is associated to the junction PS/SiO_x_/FTO on the top of the pore, in which the presence of silicon oxide at the interface allows the formation of a MIS-type diode, and the second diode *D*_2_corresponds to the interface of crystalline silicon with porous silicon (c-Si/PS).

In the characteristic *J-V* for forward bias, the current grows exponentially dominated by the diode *D*_3_ connected in parallel. *D*_3_may be attributed to the junction c-Si/SiO_x_/FTO in the bottom of the pore contact. Additionally, this model considers the presence of a voltage drop ascribed to a series resistance *Rs* and a parallel resistance *Rp*, as usually considered in a diode model. The physical model proposed is mathematically described as follows: For the two back-to-back heterojunctions, where diodes *D*_1_and *D*_2_represent the PS/SiO_x_/FTO and c-Si/PS heterojunctions, the *J-V* characteristics are

(1) $$I_{D1}=I_{l1}+I_{01}\left[\exp\left(\frac{eV_{1}}{n_{1}\mathrm{KT}}\right)-1\right]$$

(2) $$I_{D2}=-I_{l2}-I_{02}\left[\exp\left(\frac{-eV_{2}}{n_{2}\mathrm{KT}}\right)-1\right]$$

where *I*_01_, *I*_02_ and *V*_1_, *V*_2_ are the saturation currents and the voltage drops for diodes *D*_1_and *D*_2_, respectively; *I*_*l*1_ and *I*_*l*2_ are the photocurrents for diodes *D*_1_ and *D*_2_, respectively; *K* is the Boltzmann constant, and *T* is the temperature of the system. A *J-V* relation for the complete system can be found by equating the currents *I*_*D*1_ and *I*_*D*2_ through the two diodes and considering the total voltage drop as *V*_*T*_=*V*_1_ + *V*_2_. Using the additional approximation *n*=*n*_1_=*n*_2_, the total current results:

(3) $$\begin{array}{ll} I_{T}= & \left[\frac{\left(\exp(\alpha_{1})\cdot \left(\frac{I_{l2}}{I_{02}}+1\right)-\frac{I_{l1}}{I_{01}}-1\right)}{\frac{\exp(\alpha_{1})}{I_{02}}+\frac{1}{I_{01}}}\right]\\ & +\left(\frac{V_{T}-I_{T}\mathrm{Rs}}{\mathrm{Rp}}\right)+I_{03}\left[\exp(\alpha_{3})-1\right] \end{array}$$

**Figure 5:**
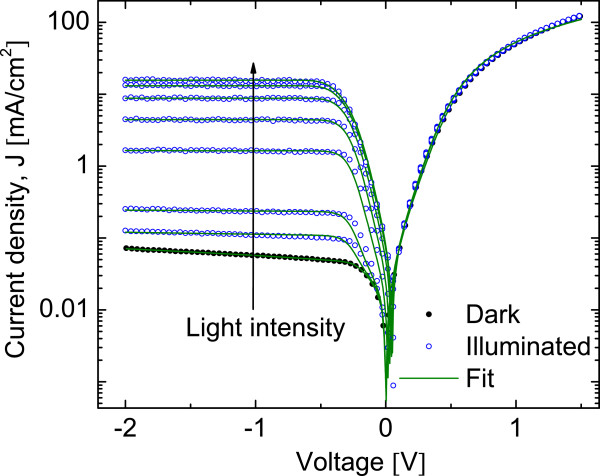
**Fit on *J-V *characteristic of PS/Ox/FTO.** Fit of characteristic *J-V* of PS/Ox/FTO on the same experimental data in Figure 3 with the model proposed in Figure 4 (Equation 3). Light intensity increases in the arrow direction ranging from dark to 110 mW/cm^2^. All parameters obtained in the dark case are used in the illuminated cases fitting only the photocurrents *I*_*l*1_and *I*_*l*2_.

In this expression, *α*_*i*_corresponds to *e*(*V*_*T*_−*I*_*T*_*Rs*)/*n*_*i*_*KT*, where *Rs* and *Rp* are series and shunt (parallel) resistances, respectively. To fit the experimental *J-V* characteristics for the PS/Ox/FTO heterojunction with expression (Equation 3), we obtain all free parameters for dark conditions (setting *I*_*l*1_=*I*_*l*2_=0). For illuminated cases, we use the parameters obtained in dark condition, and only two free parameters have been used (*I*_*l*1_ and *I*_*l*2_). Figure 5 shows the same experimental data than Figure 3 in a logarithmic scale and theoretical fitting with Equation 3.

From the fit, the ideality factor we obtain for diodes *D*_1_and *D*_2_ is *n*=1*.*6 and *n*_3_=3 for *D*_3_; the saturation currents are *I*_01_=5*.*3×10^−2^mA/cm^2^, *I*_02_=6×10^−5^ mA/cm^2^ and *I*_03_=1*.*25×10^−4^ mA/cm^2^; the serial resistance is *Rs*=43 *Ω* and parallel resistance is *Rp*=4*.*6×10^5^*Ω*. In this case, the values of *I*_0_for all the diodes are lower than obtained in the single diode of the PS/FTO heterojunction. In particular, the value of *I*_02_ is negligibly small, which prevents the current flow in forward bias on the two diode components of the circuit accordingly with the reduced photoelectric effect in the PS/Ox/FTO heterojunction. The ideality factors of diodes *D*_1_ and *D*_2_are similar to that obtained in the PS/FTO heterojunction in dark conditions, while the *n*_3_value is more similar to that obtained in the PS/FTO heterojunction in light conditions. The values of *Rs* and *Rp* are similar to the PS/FTO heterojunction.

**Figure 6:**
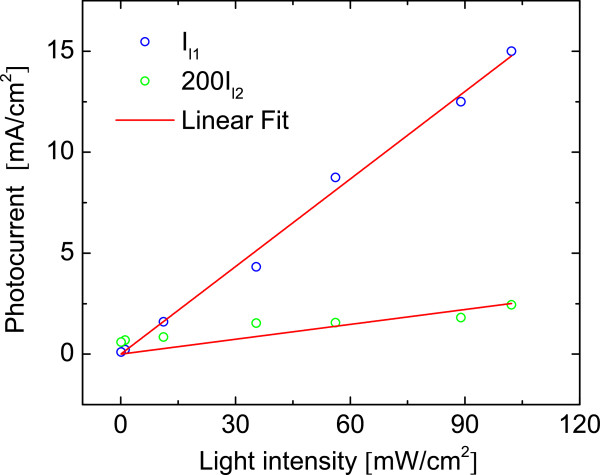
**Fit results.** Values of photocurrents *I*_*l*1_and *I*_*l*2_obtained from fitting experimental data with the model (Figure 5) as a function of illumination intensity. The values of *I*_*l*2_are scaled for a better representation. A linear fit for both parameters is also included.

Figure 6 shows the photocurrent values obtained for diodes *D*_1_ and *D*_2_ versus the light intensity for the PS/Ox/FTO heterojunction at −1*.*5 V. Both parameters show a linear dependence with light intensity, and the obtained *I*_*l*1_ is about 2,000 times greater than *I*_*l*2_, and the maximum photocurrent density, *I*_*l*1_, under reverse bias was 18 mA/cm^−2^when illuminated with 110 mW/cm^2^. This indicates that the PS/SiO_x_/FTO interface corresponding to *D*_1_ is more sensitive to illumination than *D*_2_, allowing more current to flow with increasing carrier photogeneration. This is in accordance with the fact that *D*_2_diode is in the bottom part of the heteroestructure where the light intensity is diminished by absorption and scattering in the porous layer. The position attributed to the diode *D*_3_ (also in the bottom of the heteroestructure) is in concordance to the fact that it does not present an associated photocurrent source.

## Conclusions

We prepared two types of heteroestructures, Al/c-Si/PS/SnO_2_:F and Al/c-Si/PS/SiO_x_/SnO_2_:F, on macroporous silicon substrates with high coverage of the pore walls using the spray pyrolysis technique. The *J-V* characteristics were measured in the dark and under illumination. We found that the characteristics of PS/FTO devices are well fitted using a simple diode model in both cases. Nevertheless, the fitting parameters (saturation current and ideality factor) that produce a good accordance with the experimental data are not the same in dark and illuminated conditions. This fact is an indication that this heterojunction is actually more complex than a single diode. The *J-V* characteristics of the PS/Ox/FTO heterojunction are in accordance to a more complex equivalent circuit where a second component of two back-to-back diodes is connected in parallel. Although this model incorporates three more free parameters in dark conditions, all the illumination conditions are well fitted with the same parameters obtained in the dark, where only the photocurrent intensities in the diodes are free parameters.

## Competing interests

The authors declare that they have no competing interests.

## Authors’ contributions

FAG carried out the synthesis of doped tin oxide, fabrication of porous silicon layers and *J-V* measurement of the heterojunctions. FAG, RU, LNA, RRK and RDA contributed to the conception and design of the experiments, data interpretation and writing of the manuscript. All authors discussed the results, contributed to the manuscript text, commented on the manuscript and approved its final version. All authors read and approved the final manuscript.
